# The GPR120 Agonist TUG-891 Inhibits the Motility and Phagocytosis of Mouse Alveolar Macrophages

**DOI:** 10.1155/2020/1706168

**Published:** 2020-02-20

**Authors:** Xing-Li Su, Ying-Guang Liu, Man Shi, Yan-Yan Zhao, Xiang-Yan Liang, Li-Jun Zhang, Lan-Lan Wei, Yu-Feng Zhao

**Affiliations:** Institute of Basic Medical Sciences, Xi'an Medical University, Xi'an 710021, China

## Abstract

Movement and phagocytosis characterize the fundamental actions of macrophages. Although it is known that the free fatty acid receptor GPR120 is expressed in macrophages and regulates cytokine expression to exert anti-inflammatory activities, the effects of GPR120 activation on the motility and phagocytosis of macrophages are not clear. In this study, mouse alveolar macrophages (AM) were stimulated with the GPR120 agonist TUG-891, and the changes in cell motility, intracellular Ca^2+^ concentration ([Ca^2+^]i), and the ability of phagocytosis were measured. Mouse AM in controls exhibited active movement *in vitro*, and TUG-891 significantly restrained AM movement. Meanwhile, TUG-891 stimulated a quick increase in [Ca^2+^]i in AM, which was blocked separately by the Gq protein inhibitor YM-254890, the phospholipase C (PLC) inhibitor U73122, or depletion of endoplasmic reticulum (ER) Ca^2+^ store by thapsigargin. The inhibition of AM movement by TUG-891 was eliminated by YM-254890, U73122, thapsigargin, and chelation of cytosolic Ca^2+^ by BAPTA. Moreover, TUG-891 inhibited AM phagocytosis of fluorescent microspheres, which was also blocked by YM-254890, U73122, thapsigargin, and BAPTA. In conclusion, GPR120 activation in mouse AM increases [Ca^2+^]i but inhibits the motility and phagocytosis via Gq protein/PLC-mediated Ca^2+^ release from ER Ca^2+^ store.

## 1. Introduction

G protein-coupled receptor 120 (GPR120) is one of the plasma membrane receptors for free fatty acids (FFAs) and has high binding affinity to n-3 polyunsaturated FFAs [1, 2]. It is well known that GPR120 participates in regulating body energy metabolism and immune function [3–5]. The regulation of immune function by GPR120 is mainly achieved through its action on macrophages [6]. GPR120 is highly expressed in macrophages, and its activation inhibits the expression of proinflammatory cytokines including interleukin-1 (IL-1), interleukin-6 (IL-6), and tumor necrosis factor-*α* (TNF-*α*) and promotes the expression of anti-inflammatory cytokines such as interleukin-10 (IL-10) in mouse Raw264.7 macrophages [7]. GPR120 activation also mediates the anti-inflammatory effect of n-3 polyunsaturated FFAs in human THP-1 macrophages, which is associated with the inhibition of NF*κ*B activity and cytokine production [8].

Macrophages not only release cytokines but also possess the actions of movement and phagocytosis. The motility and phagocytosis of macrophages guarantee them to remove foreign pathogens and ensure body innate immunity [9]. Phagocytosis of macrophages is a complex process that is regulated by phospholipase C (PLC) activation and the following second messengers such as diacylglycerol and Ca^2+^ [10, 11]. Macrophages possess many kinds of Ca^2+^-permeable ion channels that are located either on organelles such as endoplasmic reticulum (ER) or on the plasma membrane [12]. Gq protein activation and IP3 production can stimulate ER Ca^2+^ channel opening and increase the intracellular Ca^2+^ concentration ([Ca^2+^]i) in macrophages [13, 14]. Calcium release-activated calcium (CRAC) channels, transient receptor potential (TRP) channels, and voltage-dependent calcium channels (VDCC) are distributed on the plasma membrane of macrophages and also mediate [Ca^2+^]i increase in macrophages [15–17]. [Ca^2+^]i is linked to macrophage activities and regulates a variety of functions such as cytokine production, inflammasome formation, and phagocytosis [18–20]. GPR120 couples with Gq protein and activates the PLC signaling pathway to increase [Ca^2+^]i to stimulate glucagon-like peptide-1 (GLP-1) secretion in enteroendocrine cells [21]. The employment of this signaling pathway by GPR120 was also reported in HCT116 and HT-29 human colon epithelial cell lines [22]. Since GPR120 activation is linked to the increase in [Ca^2+^]i and [Ca^2+^]i is related to motility and phagocytosis of macrophages, it is proposed that GPR120 activation and the following PLC-stimulated signaling may regulate the motility and phagocytosis of macrophages. It is necessary to clarify the effects of GPR120 activation on the motility and phagocytosis of macrophages for the comprehensive understanding of the role of GPR120 in immune regulation.

The alveolar macrophages (AM) construct the first line of defense against inhaled particles that are deposited in the lungs and show active phagocytosis to remove the particles [23]. In addition, AM showed higher GPR120 expression than other macrophages such as peritoneal macrophages [24]. Therefore, the mouse AM in primary culture was used in this study. Long-chain FFAs, the endogenous ligands for GPR120, can be metabolized in cells, and the metabolites regulate many aspects of cell metabolism and cell signaling. Therefore, it has to distinguish the plasma membrane receptor-mediated effects from the intracellular metabolic effects when FFAs were used to activate GPR120 [25]. The potent and selective agonists for GPR120 that are not able to be metabolized in cells are helpful to facilitate understanding the physiological effects of GPR120. 3-(4-((4-Fluoro-4′-methyl-[1,1′-biphenyl]-2-yl)methoxy)phenyl) propanoic acid (TUG-891) was discovered as a potent and selective GPR120 agonist and performed regulatory actions through GPR120 *in vitro* and *in vivo* [26, 27]. In this study, TUG-891 was used to observe the effects of GPR120 activation on the movement and phagocytosis of mouse AM.

## 2. Materials and Methods

### 2.1. Materials

TUG-891 was obtained from R&D Systems Inc. (Minneapolis, MN, USA). RPMI1640 medium, fetal bovine serum (FBS), fluorescent microspheres, and Fluo-3 AM were supplied by Thermo Fisher Scientific, Inc. (Waltham, MA, USA). A rabbit anti-GPR120 antibody, a rabbit anti-F4/80 antibody, an Alexa Fluor 488-labeled goat anti-rabbit antibody, U73122, thapsigargin, and BAPTA-AM were purchased from Sigma-Aldrich (Merck KGaA; Darmstadt, Germany). YM-254890 was the product of Wako Pure Chemical Industries, Ltd. (Mie-gun, Japan). RT-PCR products were obtained from Thermo Fisher Scientific, Inc. (Waltham, MA, USA).

### 2.2. Isolation and Culture of Mouse AM

The male C57BL/6J mice at 8-10 weeks were sacrificed by CO_2_ inhalation as approved by the Animal Ethics Committee of Xi'an Medical University. The lungs were inflated by injecting 5 mL cold PBS through the bronchi, and the bronchoalveolar lavage fluid (BALF) was collected. The above procedure was repeated 3-5 times, and the total BALF was centrifuged for cell collection. The cell types in BALF were identified by immunostaining. The cells were cultured in the RPMI1640 medium containing 10% FBS in a 5% CO_2_ incubator at 37°C.

### 2.3. RT-PCR

Total RNA was extracted from BALF cells with RNA extraction kits (Takara Biotechnology Inc.) according to the manufacturer's instruction. Reverse transcription was conducted, and the cDNA was used to observe the gene expression by PCR according to the manufacturer's instruction (Takara Biotechnology Inc.). The following parameters were used for amplification: 95°C for 10 min and 32 cycles of 95°C for 5 sec, 56°C for 10 sec, and 72°C for 10 sec. The sequences of primers are as follows: mouse GPR120: 5′-GTG CCG GGA CTG GTC ATT GTG-3′ and 5′-TTG TTG GGA CAC TCG GAT CTG G-3′ and mouse GPR40: 5′-TTT CTG CCC TTG GTC ATC AC-3′ and 5′-CTA GCC ACA TTG GAG GCA TT-3′. The amplified product sizes were 340 bp and 178 bp for GPR120 and GPR40, respectively.

### 2.4. Immunostaining and Flow Cytometry Analysis

The BALF cells were fixed with 4% paraformaldehyde for 15 min and then treated with 0.3% Triton X-100 for 10 min. Then, the cells were washed with PBS and incubated with 10% goat serum for 1 h to block nonspecific binding sites. Afterwards, the cells were incubated for 1 h with an F4/80 antibody (rabbit polyclonal antibody, diluted to 1 : 1000) or a GPR120 antibody (rabbit polyclonal antibody, diluted to 1 : 1000), respectively. After being washed with PBS for 3 times, the cells were incubated for 1 h with the addition of Alexa Fluor 488-labeled secondary antibodies (goat anti-rabbit). Then, the cells were washed with PBS and incubated with DAPI to stain the nucleus. The cells were photographed under fluorescent microscopy and went through flow cytometry to analyze the rate of positive cells, respectively.

### 2.5. Cell Movement Measurement

After being cultured overnight, the mouse AM were put into the extracellular solution and recorded under transmitted light in an inverted microscope with a 20x objective (Leica DMI 6000B). The images were acquired 10 sec per image for 1 h and converted to a video at 30× speed. The movement of the cells was analyzed by ImageJ software, and the motility of macrophages was represented by the changes in the cell area and the variation of the cell area, which were used for statistical analysis. The concentration of TUG-891 used in this study was referred to the previous reports [26, 28, 29]. The concentrations of inhibitors used in this study were also referred to the reports [30–32]. The extracellular solution for cell movement measurement was composed of the following: (mmol/L) NaCl 140, KCl 4.7, CaCl_2_ 2.6, MgCl_2_ 1.2, Na_2_HPO_4_ 1.2, glucose 10, NaHCO_3_ 1, and HEPES 10 (pH = 7.4 with NaOH).

### 2.6. [Ca^2+^]i Measurement

Fluo-3 was used for the measurement of [Ca^2+^]i according to previous reports [33]. The mouse AM were cultured overnight and then incubated with Fluo-3 (2 *μ*mol/L) in the culture medium for 30 min in a 5% CO_2_ incubator at 37°C. After being washed with extracellular solution for at least 3 times, the cells were put onto the confocal microscope and the fluorescent intensity of each cell was recorded at a 6 sec interval in the XYT mode with the excitation at 488 nm (Leica SP8). The fluorescent intensity of Fluo-3 was analyzed to represent [Ca^2+^]i of macrophages. The extracellular solution used in [Ca^2+^]i measurement was the same as that used in movement measurement.

### 2.7. Phagocytosis Measurement

The method from Sharma et al. was adopted in this study [34]. The mouse AM were cultured overnight in a 5% CO_2_ incubator at 37°C and then were divided into a control group that was incubated with placebo and a TUG-891 group that was incubated with TUG-891. The fluorescent microspheres which were diluted into the medium to 1 : 50000 were added to each sample with TUG-891 or placebo. After 6 h incubation, the cells were fully washed with PBS to remove the extracellular fluorescent microspheres. The cells were detached by incubation with 0.05% trypsin for 2 min and suspended into PBS for flow cytometry analysis. The fluorescence intensity of each cell was measured to reflect the phagocytic ability of AM.

### 2.8. Statistical Analysis

Data were expressed as means ± S.E.M. The D'Agostino-Pearson omnibus test was used to test data distribution normality. The data in this study were normally distributed, and comparisons of means of multiple groups were analyzed via one-way ANOVA followed by the Bonferroni post hoc tests. *P* < 0.05 was considered significantly different.

## 3. Results

### 3.1. GPR120 Expression in Mouse AM

**Figure 1:**
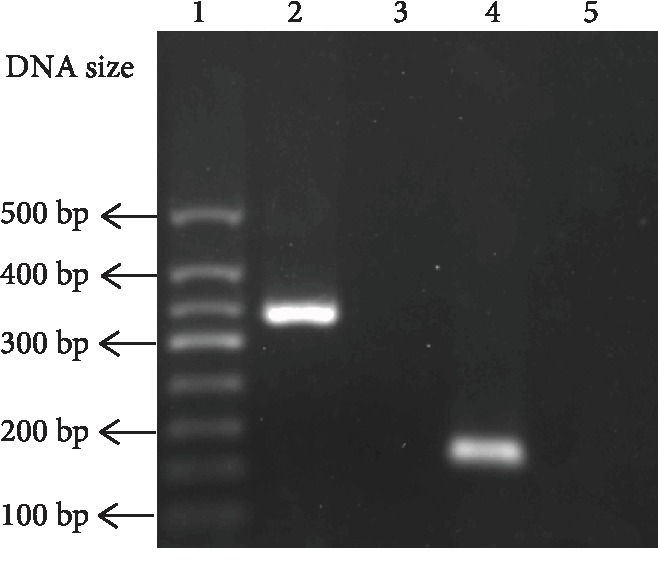
Gene expression of FFA receptors in mouse BALF cells. Lanes 1-5 were DNA marker, GPR120 in BALF cells, GPR40 in BALF cells, GPR40 in mouse islets, and the negative control to GPR120 amplification, respectively.

**Figure 2:**
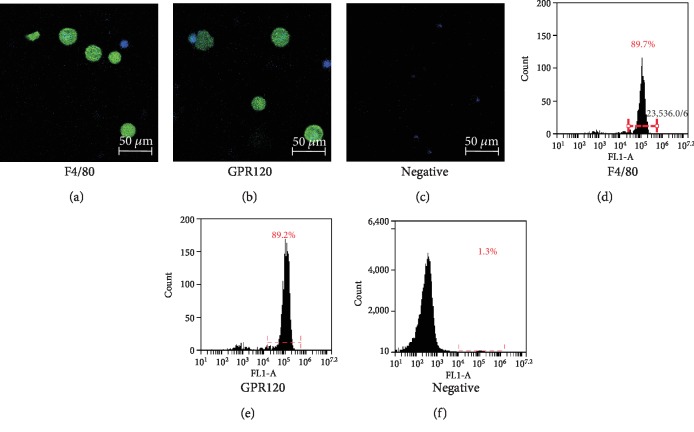
Identification and GPR120 expression in mouse alveolar macrophages. Immunostaining of mouse bronchoalveolar lavage fluid (BALF) cells with F4/80, GPR120, and negative control (a–c). Flow cytometry analysis of BALF cells after immunostaining with F4/80, GPR120, and negative control (d–f).

The cells that were harvested from the BALF for each mouse were 5 × 10^5^ ± 3.2 × 10^4^ cells (*n* = 10). As detected by RT-PCR, GPR120 was shown to be expressed in BALF cells, while another long-chain FFA receptor GPR40 was not expressed in BALF cells. The primers for GPR40 were effective and accurate as GPR40 was amplified from mouse islets, the GPR40-expressing tissue (Figure 1). Immunostaining identified that most of BALF cells were positively stained with the antibody to F4/80, the molecular marker of macrophages, indicating that most of BALF cells harvested from mice were macrophages (Figures 2(a)–2(c)). The F4/80-positive AM had a diameter of 16.34 ± 0.48 *μ*m (*n* = 18), which was larger than that of the F4/80-negative cells in BALF (10.26 ± 2.72 *μ*m, *n* = 6). Most of BALF cells were also positively stained with the GPR120 antibody. GPR120-positive cells had the diameter of 17.66 ± 0.78 *μ*m (*n* = 10) that resembled F4/80-positive macrophages. As measured by flow cytometry, the positive macrophages accounted for 84.68% ± 2.53% (*n* = 4) of the total cells and GPR120-positive cells accounted for 87.10% ± 5.14% (*n* = 4) of the total BALF cells (Figures 2(d)–2(f)).

### 3.2. The Effects of the GPR120 Agonist TUG-891 on the Motility of Mouse AM

**Figure 3:**
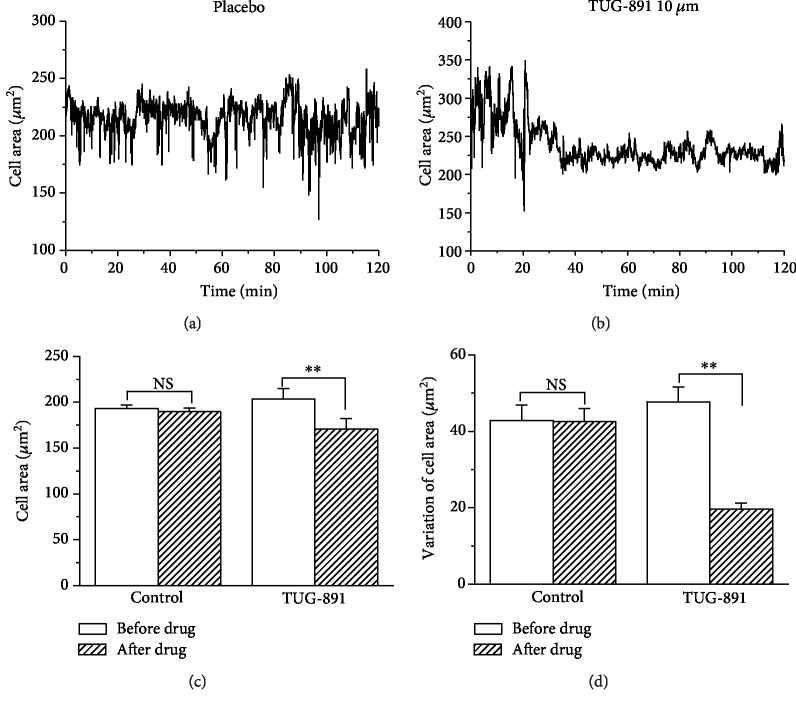
TUG-891 inhibits the movement of mouse AM. The representative changes in the cell area of macrophages in controls (a) and after TUG-891 treatment (b). The statistical analysis of the cell area (c) and the variation of changes in the cell area (d). NS means no significant difference. ^∗∗^*P* < 0.01*vs.* the control, *n* = 20.

Mouse AM in controls continued to move actively throughout the recording process in multiple modes including pseudopod stretching, morphological stretching, and migration. After being stimulated with TUG-891 (10 *μ*mol/L), the macrophages immediately tended to become round and exhibited a significant reduction in movement including pseudopod stretching, morphological stretching, and migration. The changes in the cell area and the variation of the cell area were consistent to the changes of cell movement and calculated to analyze the movement of macrophages. The cell area and the variation of the cell area were significantly decreased after TUG-891 treatment as compared to the placebo control that was treated with 0.1% DMSO (Figure 3, *P* < 0.01, *n* = 20). The video recording of the movement of macrophages could be seen in video S1 for placebo and video S2 for TUG-891 in Supplementary Materials ([Supplementary-material supplementary-material-1]).

### 3.3. The Effects of TUG-891 on [Ca^2+^]i in Mouse AM

**Figure 4:**
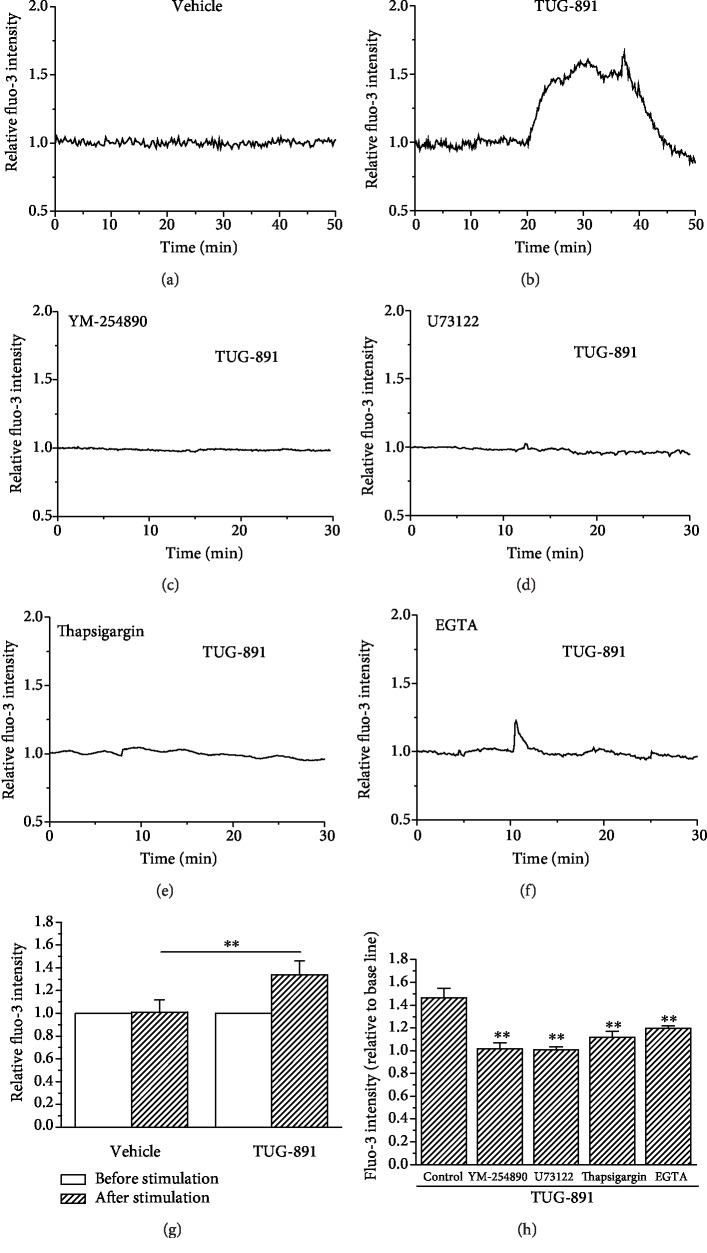
TUG-891 increases [Ca^2+^]i in mouse AM. (a–f) The representative recording of Fluo-3 fluorescent intensity in AM in controls with 0.1% DMSO vehicle, TUG-891 treatment, YM-254890 treatment+TUG-891, U73122 treatment+TUG-891, thapsigargin treatment+TUG-891, and EGTA treatment+TUG-891, respectively. The statistic of the average fluorescent intensity in macrophages in 10 min from TUG-891 or vehicle treatment is shown in (g), and the statistical analysis of inhibition of TUG-891-stimulated increase in [Ca^2+^]i by the Gq protein inhibitor YM-254890, the PLC inhibitor U-73122, depletion of Ca^2+^ store by thapsigargin, and removal of extracellular Ca^2+^ is shown in (h). ^∗∗^*P* < 0.01*vs.* the control, *n* = 20.

Fluo-3 fluorescence intensity, the indicator of [Ca^2+^]i, remained stable at the basal level throughout the recording process in the macrophages in the placebo control that was treated with 0.1% DMSO. In contrast, macrophages that were stimulated with TUG-891 (10 *μ*mol/L) showed quick increase in Fluo-3 fluorescence intensity, which remained higher than the basal levels during TUG-891 incubation and fell down after the removal of TUG-891. The profile of the changes in Fluo-3 fluorescence intensity is shown in Figures 4(a) and 4(b). Treatment of AM with YM-254890 (1 *μ*mol/L), the inhibitor of Gq protein [31], significantly suppressed TUG-891-stimulated increase in [Ca^2+^]i in AM (Figure 4(c)). U73122 (1 *μ*mol/L), the inhibitor of PLC, also significantly suppressed TUG-891-stimulated increase in [Ca^2+^]i in AM (Figure 4(d)). Thapsigargin (5 *μ*mol/L for 30 min) that inhibits the calcium pump in the ER [35] and leads to Ca^2+^ leakage and depletion of Ca^2+^ in ER Ca^2+^ store also significantly suppressed TUG-891-stimulated increase in [Ca^2+^]i in AM (Figure 4(e)). Removal of extracellular Ca^2+^ did not fully block but attenuated TUG-891-stimulated increase in [Ca^2+^]i in AM (Figure 4(f)). The changes in Fluo-3 fluorescence intensity were statistically analyzed, and it was shown that TUG-891 significantly stimulated the increase in [Ca^2+^]i in AM (Figure 4(g), *P* < 0.01, *n* = 20). The statistical analysis of the effects of the inhibitor is shown in Figure 4(h) (*P* < 0.01*vs.* control, *n* = 20). To clarify the special effect of TUG-891 on [Ca^2+^]i in AM, mouse blood lymphocytes were collected to observe the effects of TUG-891. GPR120 is not expressed in lymphocytes, and TUG-891 did not have effects on [Ca^2+^]i in lymphocytes ([Supplementary-material supplementary-material-1]).

### 3.4. The Signaling Mechanism of TUG-891-Inhibitited Movement of Mouse AM

**Figure 5:**
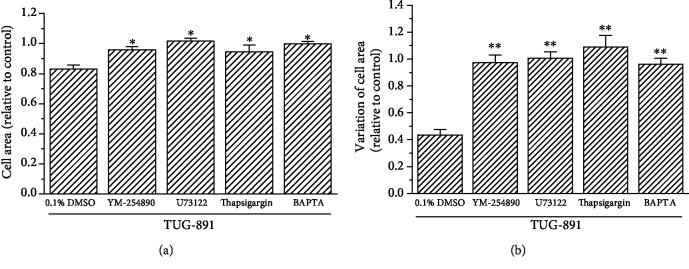
The signaling pathway for TUG-891-inhibited movement of mouse AM. AM movement was represented by the cell area and the variation of the cell area. YM-254890, U73122, thapsigargin, and BAPTA eliminated the inhibitory effects of TUG-891 on the cell area and the variation of the cell area. ^∗^*P* < 0.05*vs.* the control, *n* = 20.

YM-254890 (1 *μ*mol/L) incubation significantly suppressed the inhibitory effects of TUG-891 (10 *μ*mol/L) on macrophage movement compared with the placebo control, which was indicated by the changes in the cell area and the variation of the cell area (*P* < 0.05, *n* = 20). Treatment with U73122 (1 *μ*mol/L for 10 min) and thapsigargin (5 *μ*mol/L for 30 min) also significantly suppressed TUG-891-induced inhibition of the movement of AM as indicated by cell areas and the variation of the cell area (*P* < 0.05, *n* = 20). Chelation of cytosolic Ca^2+^ by BAPTA-AM (5 *μ*mol/L for 30 min) also significantly suppressed TUG-891-inhibited movement of macrophages (*P* < 0.05, *n* = 20). The changes in the cell area and the variation of the cell area were analyzed for each group (Figure 5).

### 3.5. The Effect of TUG-891 on Phagocytosis of Mouse AM

**Figure 6:**
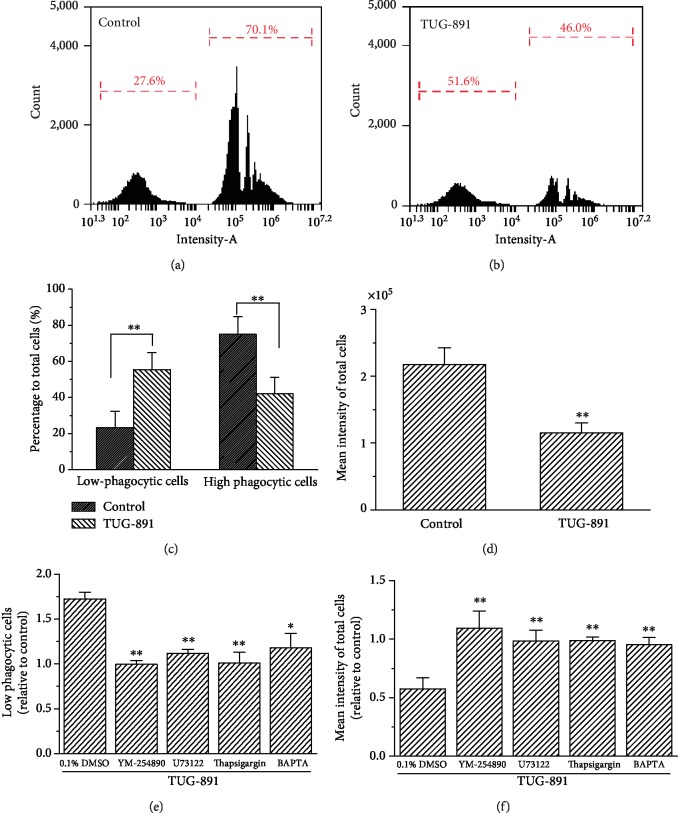
TUG-891 inhibits phagocytosis of mouse AM via the Gq protein-PLC-Ca^2+^ signaling pathway. Flow cytometry analysis of macrophages that phagocytized the fluorescent microspheres in controls (a) and after TUG-891 treatment (b). Percentage of highly and low phagocytic macrophages (c) and the average fluorescent intensity of macrophages (d). YM-254890, U-73122, thapsigargin, and BAPTA blocked the effects of TUG-891 on increase in low phagocytic cells and decrease in mean intensity of total cells (e, f). ^∗^*P* < 0.05*vs.* the control, *n* = 3.

Mouse AM in the placebo control phagocytized the fluorescent microspheres and showed increased fluorescence as measured by flow cytometry (Figure 6(a)). In contrast, the macrophages treated with TUG-891 had decreased fluorescence (Figure 6(b)). According to the intensity of the fluorescent microspheres phagocytized into the cells, the macrophages were divided into highly phagocytic macrophages with the mean fluorescence intensity of 2.53 × 10^5^ ± 0.21 × 10^5^ (74.95% ± 9.83% of total cells) and low phagocytic macrophages with the mean fluorescence intensity of 684.08 ± 46.61 (23.21 ± 9.18% of total cells). The percentage of highly phagocytic macrophages was decreased to 41.99% ± 9.15% of total cells in the TUG-891 treatment group, which was significantly lower than that of the control (*P* < 0.01, *n* = 3). In contrast, the percentage of low phagocytic macrophages increased to 55.48% ± 9.32%, showing a significant difference compared with the control (Figure 6(c), *P* < 0.01, *n* = 3). The mean fluorescence intensities of the total macrophages significantly decreased after TUG-891 treatment compared with those of the control (Figure 6(d), *P* < 0.01, *n* = 3). YM-254890 (1 *μ*mol/L), U73122 (1 *μ*mol/L for 10 min), thapsigargin (5 *μ*mol/L for 30 min), and BAPTA (5 *μ*mol/L for 30 min) all significantly suppressed the inhibitory effects of TUG-891 on phagocytosis of the macrophages as indicated by the decrease in low phagocytic macrophages and the increase in mean fluorescence intensities of the total macrophages (Figures 6(e) and 6(f), *P* < 0.05*vs.* the placebo control with 0.1% DMSO, *n* = 3).

## 4. Discussion

This study found that the GPR120 agonist TUG-891 increases [Ca^2+^]i in mouse AM but inhibits the movement and phagocytosis of mouse AM. It has been known that GPR120 activation suppresses the expression of proinflammatory cytokines and promotes the expression of anti-inflammatory cytokines in macrophages, which indicates the role of GPR120 in anti-inflammation [8, 31, 36]. The present study suggested that GPR120 exerts immunosuppressive action in macrophages by inhibiting the motility and phagocytosis besides regulating cytokine expression.

Cell movement is a dynamic process in which the skeleton proteins play a vital role [37]. The activities of skeleton proteins are regulated by a variety of intracellular signaling molecules including Ca^2+^ [38]. [Ca^2+^]i are related to pseudopod stretching and chemotactic migration of macrophages, and it is generally recognized that an appropriate fluctuation of [Ca^2+^]i is required for the movement of macrophages [39, 40]. However, due to polymorphism and complexity of the movement, the correlation of changes in [Ca^2+^]i with specific movement of macrophages and its mechanism remains to be clarified. In this study, AM contracted to the round shape with a significant decrease in the formation of pseudopods in parallel with the increase in [Ca^2+^]i when they were stimulated with TUG-891. Therefore, TUG-891-induced increase in [Ca^2+^]i did not promote the movement of macrophages, but it unexpectedly inhibited the movement of macrophages. The concurrence between [Ca^2+^]i increase and pseudopod retraction with cell area reduction was also observed in another type of macrophages, the osteoclast, when it was stimulated with a platelet-activating factor [41, 42]. The reasons for the inhibition of macrophage motility after an intense increase in [Ca^2+^]i are not clear. An appropriate rise of [Ca^2+^]i with local oscillation in a certain pattern ensured the normal movement and phagocytosis of the macrophages. It is proposed that the intense rising of [Ca^2+^]i that was stimulated with TUG-891 led to continuous strong contraction of skeleton proteins in macrophages, which causes their inability to free stretch and consequently restrained AM movement.

Previous studies showed that GPR120 acts through Gq protein to activate PLC and the following DAG/IP_3_ signaling molecules, which stimulated Ca^2+^ release from the ER and lead to PKC activation. This signaling pathway was related to GPR120 agonist-stimulated secretion of GLP-1 from enteroendocrine cells [21]. In this study, the Gq protein inhibitor YM-254890 and the PLC inhibitor U73122 suppressed the increase in [Ca^2+^]i as well as the inhibition of macrophage movement that was induced by TUG-891. It indicates that TUG-891 stimulates the increase in [Ca^2+^]i in AM through Gq protein to activate PLC and the following phosphatidylinositol signaling pathway. In addition, the depletion of intracellular Ca^2+^ store of ER by thapsigargin eliminated TUG-891-stimulated increase in [Ca^2+^]i in macrophages. Therefore, this study indicates that TUG-891 stimulated the increase in [Ca^2+^]i through Gq protein and Ca^2+^ mobilization of intracellular Ca^2+^ stores in mouse AM. On the other hand, removal of extracellular Ca^2+^ attenuated TUG-891-stimulated increase in [Ca^2+^]i in macrophages. It is suggested that Ca^2+^ release triggered Ca^2+^ influx through the plasma membrane. Ca^2+^ influx through the plasma membrane is mediated by variable Ca^2+^-permeable ion channels, and the specific ion channels need to be investigated in the future study.

As shown by the phagocytosis of fluorescent microspheres, AM in this study exhibited phagocytic ability, which is exactly consistent with previous reports [43, 44]. TUG-891 significantly inhibited AM phagocytosis of fluorescent microspheres. Both phagocytosis and cell motility of AM are controlled by the rearrangement of microfilaments such as actin and actin-binding proteins [45]. The phagocytosis of macrophages is a highly complicated process and closely correlated with their movement. The inhibition of the motility of macrophages may be the reason for the inhibition of phagocytosis. TUG-891-inhibited phagocytosis was also suppressed by the treatment with YM-254890, U73122, and thapsigargin and the chelation of cytosolic Ca^2+^ by BAPTA. It is suggested that TUG-891 activated Gq/PLC-DAG/IP3-Ca^2+^ signaling to inhibit phagocytosis of AM.

TUG-891 was used in this study to activate GPR120 for two reasons. First, TUG-891 is a more selective and potent agonist for GPR120 than GW9508 and NCG21 [27, 46]. Second, TUG-891 is not able to be metabolized in cells to regulate cell function via metabolites, and it avoided the disadvantage of FFAs that can regulate cell function through intracellular metabolites [25, 47]. Although TUG-891 is able to activate GPR40, mouse AM do not express GPR40 as demonstrated by RT-PCR in this study. Therefore, the effects of TUG-891 on mouse AM were probably mediated by GPR120. A recent study showed that TUG-891 leads to a reduction in mouse body weight and fat mass and increase in brown adipocyte metabolism, which is GPR120-dependent since TUG-891 lost the effects in GPR120-knockout mice [26]. This supports that TUG-891 takes effects by activating GPR120. Therefore, it indicates that TUG-891 inhibits the motility and phagocytosis of mouse AM through GPR120 activation and the Gq protein-coupled PLC-DAG/IP3 signaling pathway in mouse AM.

GPR120 activation-induced inhibition of AM phagocytosis possibly is involved in maintaining a nonactivating state of macrophages to avoid tissue inflammatory damage. It was found that lipopolysaccharide, the indicator of bacterial infection, inhibited GPR120 expression in macrophages [48]. Here, we propose that inhibition of GPR120 expression under infection inflammation may strengthen the reaction to pulmonary infection and facilitate the elimination of infection [49, 50]. On the other hand, GPR120 activation improves chronic noninfectious inflammation in adipose tissue of obese mice [5, 51, 52]. It is suggested that GPR120 is not inhibited in expression and may exhibit anti-inflammatory effects under noninfectious inflammation. The GPR120 agonist is expected to take therapeutic effect for pulmonary noninfectious inflammation. Nevertheless, the pharmacological implications of GPR120 agonists or antagonists on the therapy of pulmonary diseases need to be studied in the future.

## Data Availability

The data to support the findings of this study are available from the corresponding author upon request.

## Supplementary Materials

Supplementary Materials Video S1-macrophage movement-placebo: mouse AM in controls continued to move actively throughout the recording process and placebo treatment (0.1% DMSO) did not influence the movement. Video S2-macrophage movement-TUG-891: TUG-891 (10 mmol/L) treatment resulted in the reduction in movement with the tendency to become round in mouse AM cells. Supplement 2: the nonexpression of GPR120 in mouse blood lymphocytes and the ineffectiveness of TUG-891 on [Ca^2+^] in lymphocytes.

## References

[1] Milligan G., Alvarez-Curto E., Hudson B. D., Prihandoko R., Tobin A. B. (2017). FFA4/GPR120: pharmacology and therapeutic opportunities. *Trends in Pharmacological Sciences*.

[2] Rayasam G. V., Tulasi V. K., Davis J. A., Bansal V. S. (2007). Fatty acid receptors as new therapeutic targets for diabetes. *Expert Opinion on Therapeutic Targets*.

[3] Greenhill C. (2014). Gpr120 agonist has anti-inflammatory and insulin-sensitizing effects. *Nature Reviews. Endocrinology*.

[4] McLarnon A. (2012). GPR120 dysfunction can cause obesity in mice and humans. *Nature Reviews Gastroenterology & Hepatology*.

[5] Song T., Yang Y., Zhou Y., Wei H., Peng J. (2017). GPR120: a critical role in adipogenesis, inflammation, and energy metabolism in adipose tissue. *Cellular and Molecular Life Sciences*.

[6] Im D. S. (2016). Functions of omega-3 fatty acids and FFA4 (GPR120) in macrophages. *European Journal of Pharmacology*.

[7] Oh D. Y., Talukdar S., Bae E. J. (2010). GPR120 is an omega-3 fatty acid receptor mediating potent anti-inflammatory and insulin-sensitizing effects. *Cell*.

[8] Williams-Bey Y., Boularan C., Vural A. (2014). Omega-3 free fatty acids suppress macrophage inflammasome activation by inhibiting NF-*κ*B activation and enhancing autophagy. *PLoS One*.

[9] Coleman D. L. (1986). Regulation of macrophage phagocytosis. *European Journal of Clinical Microbiology*.

[10] Dewitt S., Hallett M. B. (2002). Cytosolic free Ca(2+) changes and calpain activation are required for beta integrin-accelerated phagocytosis by human neutrophils. *The Journal of Cell Biology*.

[11] Myers J. T., Swanson J. A. (2002). Calcium spikes in activated macrophages during Fcgamma receptor-mediated phagocytosis. *Journal of Leukocyte Biology*.

[12] Feske S., Wulff H., Skolnik E. Y. (2015). Ion channels in innate and adaptive immunity. *Annual Review of Immunology*.

[13] Mayne M., Holden C. P., Nath A., Geiger J. D. (2000). Release of calcium from inositol 1,4,5-trisphosphate receptor-regulated stores by HIV-1 Tat regulates TNF-*α* production in human macrophages. *Journal of Immunology*.

[14] Layhadi J. A., Fountain S. J. (2017). Influence of ER leak on resting cytoplasmic Ca_2+_ and receptor-mediated Ca_2+_ signalling in human macrophage. *Biochemical and Biophysical Research Communications*.

[15] Antony C., Mehto S., Tiwari B. K., Singh Y., Natarajan K. (2015). Regulation of L-type voltage gated calcium channel CACNA1S in macrophages upon Mycobacterium tuberculosis infection. *PLoS One*.

[16] Sharma D., Tiwari B. K., Mehto S. (2016). Suppression of protective responses upon activation of L-type voltage gated calcium channel in macrophages during *Mycobacterium bovis* BCG infection. *PLoS One*.

[17] Molteni L., Rizzi L., Bresciani E. (2018). STIM proteins and Orai Ca^2+^ channels are involved in the intracellular pathways activated by TLQP-21 in RAW264.7 macrophages. *Frontiers in Pharmacology*.

[18] Link T. M., Park U., Vonakis B. M., Raben D. M., Soloski M. J., Caterina M. J. (2010). TRPV2 has a pivotal role in macrophage particle binding and phagocytosis. *Nature Immunology*.

[19] Demaurex N., Nunes P. (2016). The role of STIM and ORAI proteins in phagocytic immune cells. *American Journal of Physiology. Cell Physiology*.

[20] Li M., Fang X. Z., Zheng Y. F. (2019). Transient receptor potential vanilloid 4 is a critical mediator in LPS mediated inflammation by mediating calcineurin/NFATc3 signaling. *Biochemical and Biophysical Research Communications*.

[21] Hirasawa A., Tsumaya K., Awaji T. (2005). Free fatty acids regulate gut incretin glucagon-like peptide-1 secretion through GPR120. *Nature Medicine*.

[22] Kim J. M., Lee K. P., Park S. J. (2015). Omega-3 fatty acids induce Ca(2+) mobilization responses in human colon epithelial cell lines endogenously expressing FFA4. *Acta Pharmacologica Sinica*.

[23] Hiraiwa K., van Eeden S. F. (2013). Contribution of lung macrophages to the inflammatory responses induced by exposure to air pollutants. *Mediators of Inflammation*.

[24] Liu Y., Zhao Y., Wei L. (2018). Differential expression and correlation to cytokine expressions of FFAR4 in peritoneal and alveolar macrophages. *Chinese Journal of Cellular and Molecular Immunology*.

[25] Zhao Y., Wang L., Qiu J., Zha D., Sun Q., Chen C. (2013). Linoleic acid stimulates [Ca2+]i increase in rat pancreatic beta-cells through both membrane receptor- and intracellular metabolite-mediated pathways. *PLoS One*.

[26] Schilperoort M., Dam A. D., Hoeke G. (2018). The GPR120 agonist TUG-891 promotes metabolic health by stimulating mitochondrial respiration in brown fat. *EMBO Molecular Medicine*.

[27] Hudson B. D., Shimpukade B., Mackenzie A. E. (2013). The pharmacology of TUG-891, a potent and selective agonist of the free fatty acid receptor 4 (FFA4/GPR120), demonstrates both potential opportunity and possible challenges to therapeutic agonism. *Molecular Pharmacology*.

[28] Anbazhagan A. N., Priyamvada S., Gujral T. (2016). A novel anti-inflammatory role of GPR120 in intestinal epithelial cells. *American Journal of Physiology. Cell Physiology*.

[29] Gao B., Huang Q., Jie Q. (2015). GPR120: a bi-potential mediator to modulate the osteogenic and adipogenic differentiation of BMMSCs. *Scientific Reports*.

[30] Bleasdale J. E., Thakur N. R., Gremban R. S. (1990). Selective inhibition of receptor-coupled phospholipase C-dependent processes in human platelets and polymorphonuclear neutrophils. *The Journal of Pharmacology and Experimental Therapeutics*.

[31] Nishimura A., Kitano K., Takasaki J. (2010). Structural basis for the specific inhibition of heterotrimeric Gq protein by a small molecule. *Proceedings of the National Academy of Sciences of the United States of America*.

[32] Quynh Doan N. T., Christensen S. B. (2015). Thapsigargin, origin, chemistry, structure-activity relationships and prodrug development. *Current Pharmaceutical Design*.

[33] Zhao Y. F., Xu R., Hernandez M., Zhu Y., Chen C. (2003). Distinct intracellular Ca^2+^ response to extracellular adenosine triphosphate in pancreatic *β*-cells in rats and mice. *Endocrine*.

[34] Sharma L., Wu W., Dholakiya S. L. (2014). Assessment of phagocytic activity of cultured macrophages using fluorescence microscopy and flow cytometry. *Methods in Molecular Biology*.

[35] Thomas D., Hanley M. R. (1994). Pharmacological tools for perturbing intracellular calcium storage. *Methods in Cell Biology*.

[36] Yamada H., Umemoto T., Kakei M. (2017). Eicosapentaenoic acid shows anti-inflammatory effect via GPR120 in 3T3-L1 adipocytes and attenuates adipose tissue inflammation in diet-induced obese mice. *Nutrition & Metabolism*.

[37] Eddy R. J., Pierini L. M., Matsumura F., Maxfield F. R. (2000). Ca2+-dependent myosin II activation is required for uropod retraction during neutrophil migration. *Journal of Cell Science*.

[38] Bengtsson T., Jaconi M. E., Gustafson M. (1993). Actin dynamics in human neutrophils during adhesion and phagocytosis is controlled by changes in intracellular free calcium. *European Journal of Cell Biology*.

[39] Nunes P., Demaurex N. (2010). The role of calcium signaling in phagocytosis. *Journal of Leukocyte Biology*.

[40] Melendez A. J., Tay H. K. (2008). Phagocytosis: a repertoire of receptors and Ca(2+) as a key second messenger. *Bioscience Reports*.

[41] Wood D. A., Hapak L. K., Sims S. M., Dixon S. J. (1991). Direct effects of platelet-activating factor on isolated rat osteoclasts. Rapid elevation of intracellular free calcium and transient retraction of pseudopods. *The Journal of Biological Chemistry*.

[42] Lapierre D. M., Tanabe N., Pereverzev A. (2010). Lysophosphatidic acid signals through multiple receptors in osteoclasts to elevate cytosolic calcium concentration, evoke retraction, and promote cell survival. *The Journal of Biological Chemistry*.

[43] Schrijvers D. M., Martinet W., De Meyer G. R., Andries L., Herman A. G., Kockx M. M. (2004). Flow cytometric evaluation of a model for phagocytosis of cells undergoing apoptosis. *Journal of Immunological Methods*.

[44] Leclerc L., Boudard D., Pourchez J. (2010). Quantification of microsized fluorescent particles phagocytosis to a better knowledge of toxicity mechanisms. *Inhalation Toxicology*.

[45] Rougerie P., Miskolci V., Cox D. (2013). Generation of membrane structures during phagocytosis and chemotaxis of macrophages: role and regulation of the actin cytoskeleton. *Immunological Reviews*.

[46] Shimpukade B., Hudson B. D., Hovgaard C. K., Milligan G., Ulven T. (2012). Discovery of a potent and selective GPR120 agonist. *Journal of Medicinal Chemistry*.

[47] Zhao Y. F., Pei J., Chen C. (2008). Activation of ATP-sensitive potassium channels in rat pancreatic beta-cells by linoleic acid through both intracellular metabolites and membrane receptor signalling pathway. *The Journal of Endocrinology*.

[48] Zhao Y. Y., Fu H., Liang X. Y. (2019). Lipopolysaccharide inhibits GPR120 expression in macrophages via Toll-like receptor 4 and p38 MAPK activation. *Cell Biology International*.

[49] Alber A., Howie S. E., Wallace W. A., Hirani N. (2012). The role of macrophages in healing the wounded lung. *International Journal of Experimental Pathology*.

[50] Belchamber K. B. R., Donnelly L. E. (2017). Macrophage dysfunction in respiratory disease. *Results and Problems in Cell Differentiation*.

[51] Ichimura A., Hirasawa A., Poulain-Godefroy O. (2012). Dysfunction of lipid sensor GPR120 leads to obesity in both mouse and human. *Nature*.

[52] Oh D. Y., Walenta E. (2014). The role of omega-3 fatty acid receptor GPR120 in insulin resistance. *International Journal of Obesity Supplements*.

