# Supplementary figures and images for: The GPR120 Agonist TUG-891 Inhibits the Motility and Phagocytosis of Mouse Alveolar Macrophages
Source: Biomed Res Int. 2020 Feb 20;2020:1706168. doi: 10.1155/2020/1706168 (PMC7056993; doi:10.1155/2020/1706168)

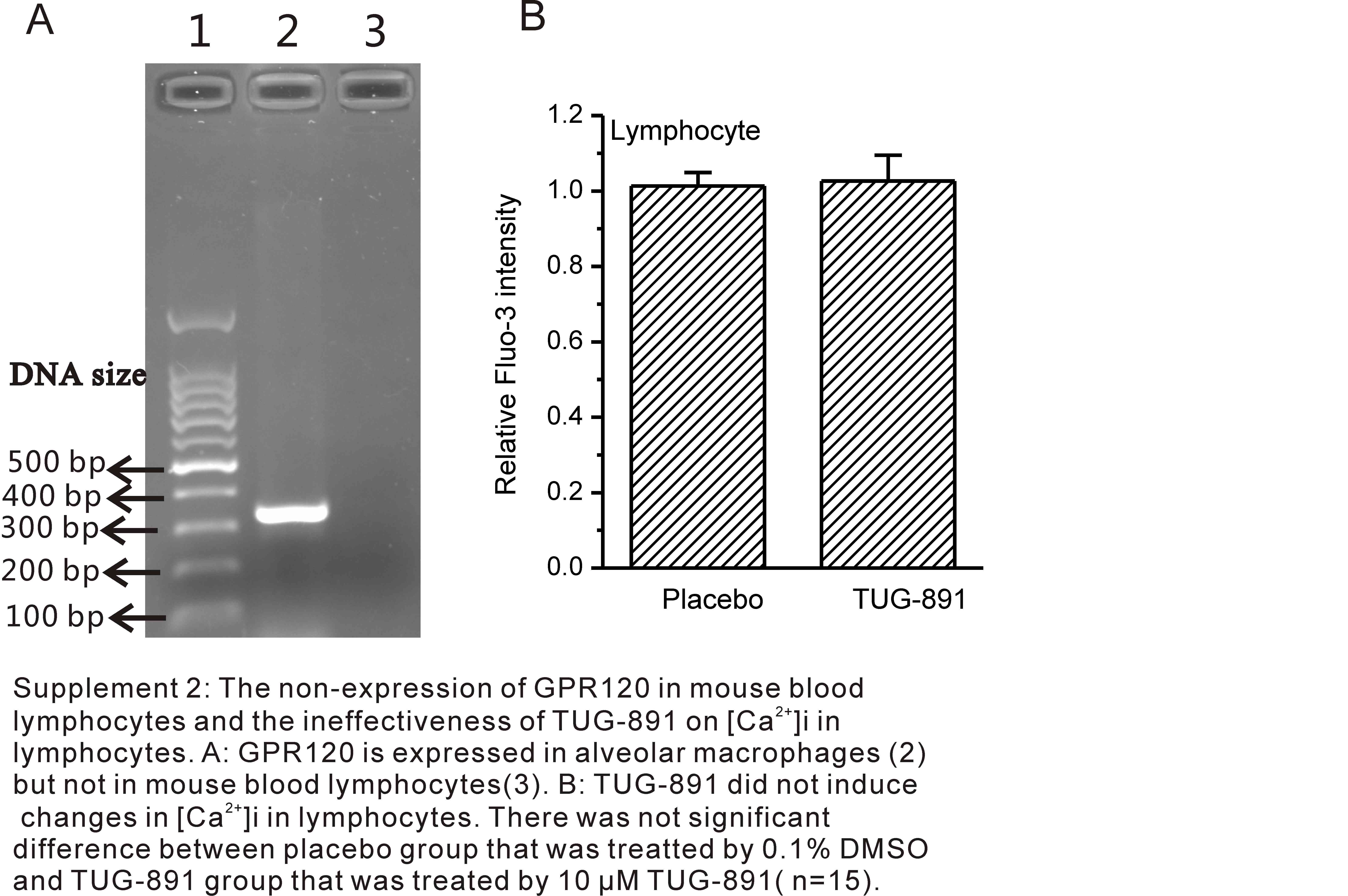

Supplement: Supplementary Materials — Video S1-macrophage movement-placebo: mouse AM in controls continued to move actively throughout the recording process and placebo treatment (0.1% DMSO) did not influence the movement. Video S2-macrophage movement-TUG-891: TUG-891 (10 mmol/L) treatment resulted in the reduction in movement with the tendency to become round in mouse AM cells. Supplement 2: the nonexpression of GPR120 in mouse blood lymphocytes and the ineffectiveness of TUG-891 on [Ca2+] in lymphocytes. [file 1706168.f1.zip › mat.1706168.v2.jpg]
